# Comparative Evaluation of Shear Bond Strength, Enamel Microfracture, and Adhesive Remnant Index of APC Flash-Free System Versus Traditional Bonding: An In-Vitro Study

**DOI:** 10.7759/cureus.60928

**Published:** 2024-05-23

**Authors:** Alok Ranjan, Waseem Khan, Poonam Achalkar, Ankita Pawar, Dilip Makdum, Viraj Kharkhar

**Affiliations:** 1 Orthodontics, Bharati Vidyapeeth (Deemed to be University) Dental College and Hospital, Navi Mumbai, IND; 2 Orthodontics, Serene Dental Clinic, Pune, IND; 3 Orthodontics, Bharati Vidyapeeth (Deemed to be University) Dental College and Hospital, Pune, IND; 4 Oral Pathology, Bharati Vidyapeeth (Deemed to be University) Dental College and Hospital, Sangli, IND; 5 Oral and Maxillofacial Surgery, Bharati Vidyapeeth (Deemed to be University) Dental College and Hospital, Navi Mumbai, IND

**Keywords:** traditional brackets, apc flash free, adhesive remnant index, enamel microfracture, shear bond strength

## Abstract

Introduction: In orthodontics, shear bond strength plays an important role because it provides a good bond between the brackets and tooth surface; it avoids fracture of the tooth surface and prevents debonding of brackets from the tooth surface. All of these allow sufficient treatment time. Many factors, including the adhesive, its thickness, its strength, the bonding procedure, the clinician's ability, the base design, the geometry of the bracket, the material, and the kind of bracket all contribute to the shear bond strength. Brackets joined using conventional adhesive and adhesive pre-coated (APC) flash-free glue were the subjects of this comparison and evaluation research, which aimed to measure shear bond strength, enamel microfracture, and adhesive residual index.

Method: 60 recently removed premolars from humans were used in this investigation. Before mounting on the acrylic block, the teeth were meticulously cleaned and preserved in artificial saliva. Two groups were formed from the collected premolars the control group and the experimental group. For the control group, we used American Orthodontics (AO) Master/Mini Master series brackets glued with resin composite kits. To make sure the adhesive was uniformly thick, we flashed extra adhesive around the brackets. In the meantime, samples were bonded using 3M Unitek APC flash-free technology in the experimental group.

Results: The research indicated that there was a statistically significant difference between the two groups to the adhesive remnant index (ARI) and mean shear bond strength. The shear bond strength of the experimental group averaged 10.96 megapascals (MPa), whereas the control group's was 5.70 MPa. The control group's ARI score was 2.97, whereas the experimental group's score was 2.4. There was no statistically significant change seen in enamel microfracture.

Conclusion: A more robust shear bond may be possible using APC flash-free brackets. Compared to conventional bonding techniques and brackets, APC flash-free brackets have a lower adhesive residual index. The APC flash-free bracket technology also causes more enamel microfracture than conventional bonding and bracketing methods.

## Introduction

In orthodontics, shear bond strength plays an important role. The good bonding between brackets and tooth surface avoids fracture of tooth surface and prevents debonding of brackets from the tooth surface, increasing treatment durability. Shear bond strength is mainly dependent on the design of the bracket base, geometry of the bracket, material, and type of bracket, the adhesive used, thickness of adhesive used, strength of the adhesive, bonding technique, and skill of the clinician. In 1975, Reynolds suggested that shear bond strength should be 5-7 megapascals (MPa), ideal for carrying out orthodontic tooth movement [1].

The idea of attaching different resins to enamel surfaces has led to the development of applications in all sectors of dentistry since Buonocore first the acid-etched bonding process in 1955. One of these applications is the gluing of orthodontic brackets to tooth surfaces. The benefits of this method over banding are numerous: the patient is better able to remove plaque, soft tissues are less irritated, there are no posttreatment band spaces, partially erupted teeth can be attached more easily, the risk of decalcification from a loose band is reduced, caries can be more easily detected and treated, and the patient looks better overall. Enamel decalcification around orthodontic brackets is a major issue that clinicians still face, even with all these benefits. This is especially true in patients with less than ideal oral hygiene or once white spot lesions have formed after debonding.

White spot lesions impacted 97% of individuals undergoing fixed orthodontic treatment, according to a 2005 study by Boersma et al. [2]. Total isolation is required for composite bonding, making it more technique-sensitive than resin-modified glass ionomer cement bonding. Additionally, no saliva should be present on the bonding surface. Research by Sharma et al. indicates that resin-modified glass ionomer cement outperforms its traditional counterpart in terms of mechanical and physical qualities [3]. Its cohesive strength is higher than that of glass ionomer cement, while its modulus of elasticity is lower. Conventional glass ionomer cement does not attach as strongly as resin-modified glass ionomer cement does to the tooth. Furthermore, its binding strength surpasses that of resin composite. [3].

The orthodontist has access to a vast array of cements, including those that are light-activated, chemical-cured, variably filled, and others. The primary objective is to prevent white spot lesions or cavities from developing under and around the bracket by creating an adequate marginal seal with little bonding material [4]. Before selecting an adhesive for orthodontic bracket bonding, clinicians should consider the bond quality, the mechanism of failure upon debonding, and the convenience of cleaning up adhesive remnants after debonding. As a good bonding material, orthodontists always require less flash to avoid iatrogenic disadvantages such as plaque accumulation. According to Grünheid et al., there will no longer be any mess to clean up after bonding orthodontic brackets thanks to the new flash-free glue [5]. Less flash removal is required when using an adhesive pre-coated (APC) flash-free bracket, which has adhesive already applied to the brackets. To ensure that the brackets stay affixed to the tooth for the duration of fixed appliance orthodontic treatment, a high-quality bond is necessary. There should be no more than a few tiny holes in the adhesive layer for the bond to remain strong and prevent white spot lesions from forming. If you want to take out a bonded bracket, the bonding material has to fail somewhere: at the bracket-to-bonding-material interface, at the bonding material's internal interface, or at the enamel surface contact. Because the bonding material could rip the enamel surface as it pulls away from the tooth, debonding shouldn't fail at the enamel surface once a solid attachment to the enamel has been made. Since this is the case, the bonding substance's interaction with the bracket is often seen as the weakest link by orthodontists. It would be great if, after debonding, the adhesive stayed on the tooth. Any remaining glue on the tooth surface has to be removed [5].

Failure at adhesive enamel interface causes enamel loss. Debonding sessions are notorious as one of the lengthiest in orthodontic treatments due in large part to the amount of time needed to remove the residual adhesive. Longer appointments are costly for the doctor and waste the time of the patient. The mesh-base brackets were found to be more retentive in tension by Thanos et al., whereas the metal-base brackets were shown to be more retentive in shear [6].

The APC flash-free adhesive bracket, because of its ability to bond even in saliva’s presence, can be a good bracket with bonding agent during orthodontic bonding procedure. Orthodontists used to have to use a positioning tool or dental probe to scoop out any leftover resin bonding material before curing the bracket, but that's all changed now. 3M Unitek (Monrovia, USA) announced the APC flash-free technology (APC Flash-Free Adhesive Coated Appliance System) in 2014, which ostensibly did away with the requirement to remove surplus material. Any orthodontic bracket base may be used to create the system, which is made possible by using a resin-soaked nonwoven mat. When the low-viscosity, see-through resin is put into the enamel, it produces a channeling border around the bracket's periphery. Proposed benefits of this adhesive system include eliminating the need for adhesive clean-up, speeding up the bonding and placing of brackets, and enhancing the capacity to focus on the positioning process [7-9].

When choosing an adhesive to attach orthodontic brackets, it's crucial to consider bond quality, debonding failure mechanism, and how easy it will be to clean up adhesive remnants. Success or failure for the new glue will depend on physician choice and acceptance. Therefore, this study set out to evaluate the APC flash-free bracket system to traditional bonding methods in terms of shear bond strength, adhesive remnant index (ARI), and enamel microfracture. Nevertheless, the primary objective was to produce an adequate marginal seal with little bonding material surrounding the bracket in order to prevent white spot lesions or caries from developing beneath and around the bracket.

## Materials and methods

A total of 60 newly removed premolars were used in the investigation. As a component of orthodontic therapy, the teeth were removed in the Oral Surgery Department. Ethical approval was taken from the Vasantdada Patil Dental College and Hospital Institutiona Review Board with the reference number Vspct/Vpdch/177/19-20. The root surfaces of all teeth were cleared of soft tissue debris and blood. At room temperature, the samples were preserved in synthetic saliva. Before the brackets were attached, the teeth were immersed in fake saliva.

Inclusion criteria included freshly extracted human premolars with intact buccal surface and no caries. Additionally, they should not have been previously treated with hydrogen peroxide. Last, the extracted human premolars should not have been stored for more than 60 days. Exclusion criteria included teeth with caries or cracked buccal surfaces.

**Figure 1:**
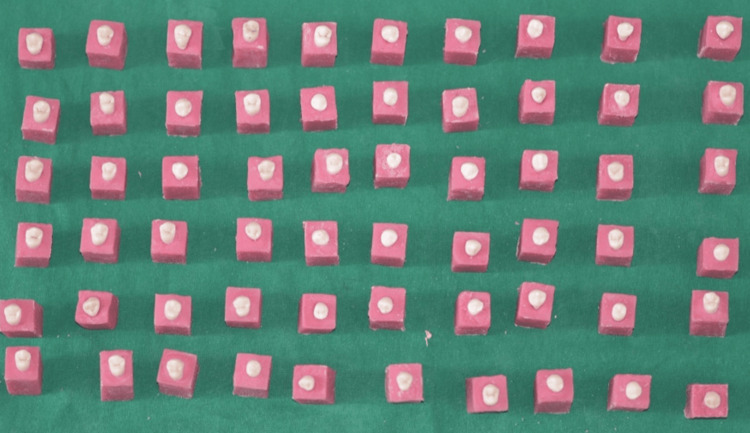
Teeth mounted in acrylic blocks

Once extracted, the premolars were rinsed and preserved in artificial saliva for a period ranging from three days to sixty days at room temperature. They were mounted on an acrylic block, keeping the crown surface open and the root fixed into the acrylic block. Specimens were then divided into two groups (30 premolars in each group): control and experimental. Teeth in the two groups were cleaned with normal saline (Figure 1).

**Figure 2:**
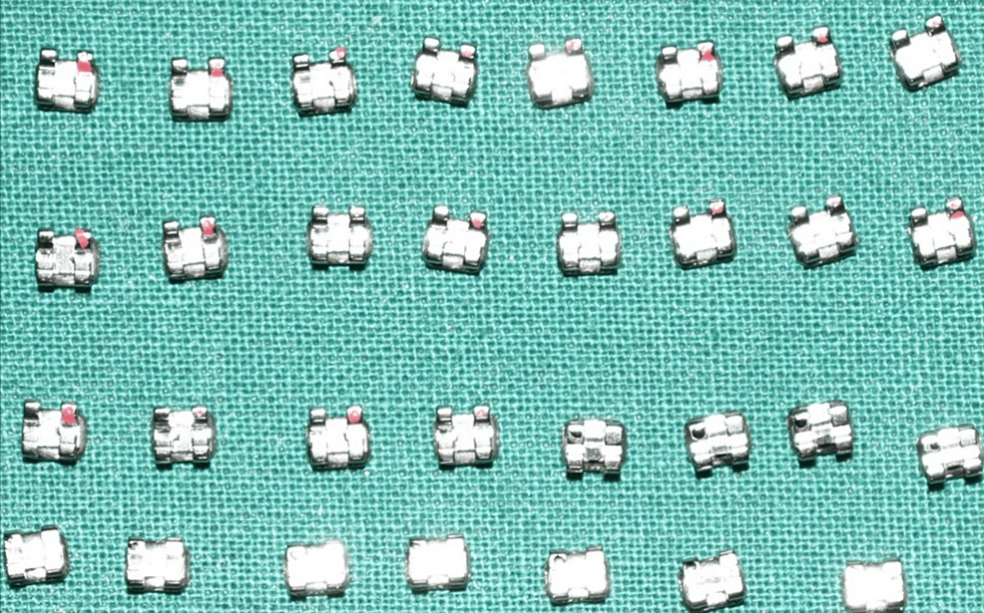
Standard premolar brackets (AO Master/Mini Master series) AO:  American Orthodontics

After that, the two groups underwent the following process. For one minute, using a micro brush, specimens in Group I, the control group, were bonded with orthodontic brackets from the American Orthodontics (AO) Master/Mini Master series resin composite kit. The specimens were then cleaned and dried before being acid-etched for 30 seconds with 37% phosphoric acid. After that, a micro brush was used to apply a thin coat of primer, which was then light-cured for 20 seconds. The whole base of the bracket was coated with composite resin. Empty spaces or bubbles were sidestepped. A homogeneous thickness of adhesive was achieved by placing the bracket on the tooth surface with enough force to create a flash of extra glue around it. Next, we used a scaler to remove any extra glue. After that, we dried it under a halogen light for 40 seconds (10 seconds on each side) (Figure 2).

**Figure 3:**
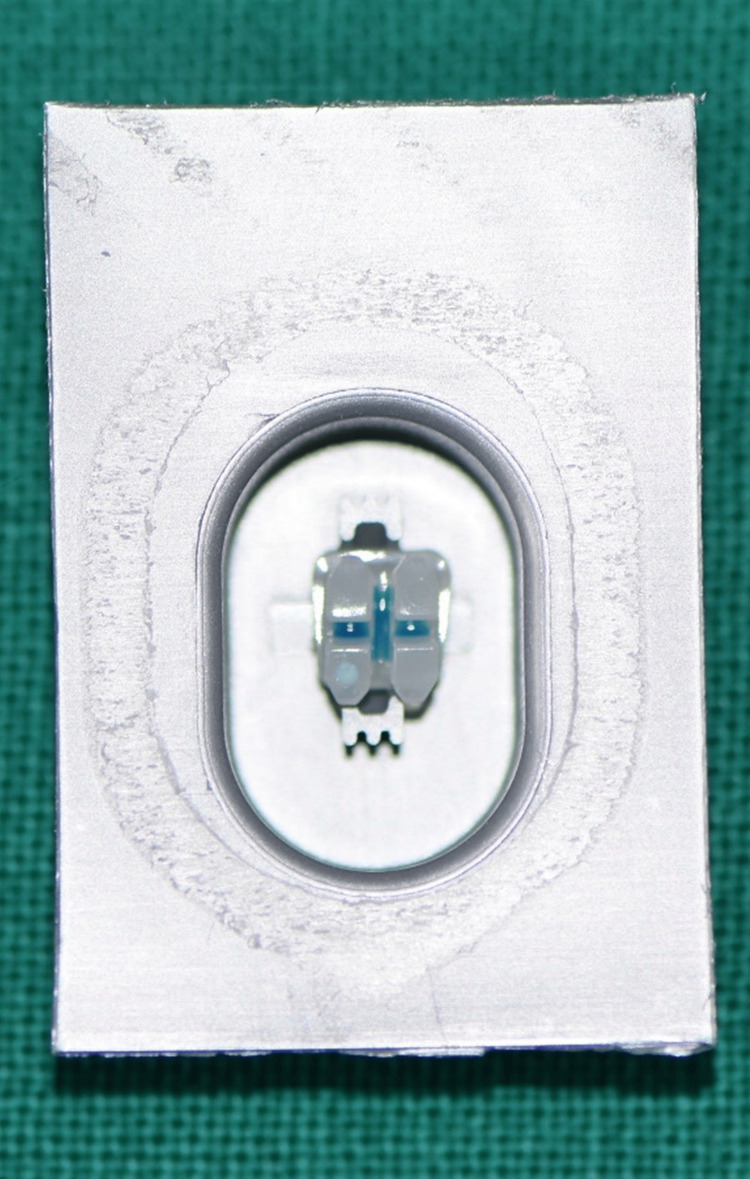
APC flash-free adhesive coated bracket APC: Adhesive pre-coated

30 premolar specimens were bonded in Group II of the experiment using 3M Unitek's APC Flash-Free Adhesive Coated Appliance System. This appliance solution eliminates the need to remove any more material by using APC flash-free technology. Using a nonwoven mat, the system was placed to the orthodontic bracket base during manufacture, and saturated adhesive resin was obtained. When the thin, low-viscosity resin was applied to the enamel surface, it formed a channeling border around the bracket's edge (Figure 3).

Laboratory procedure

**Figure 4:**
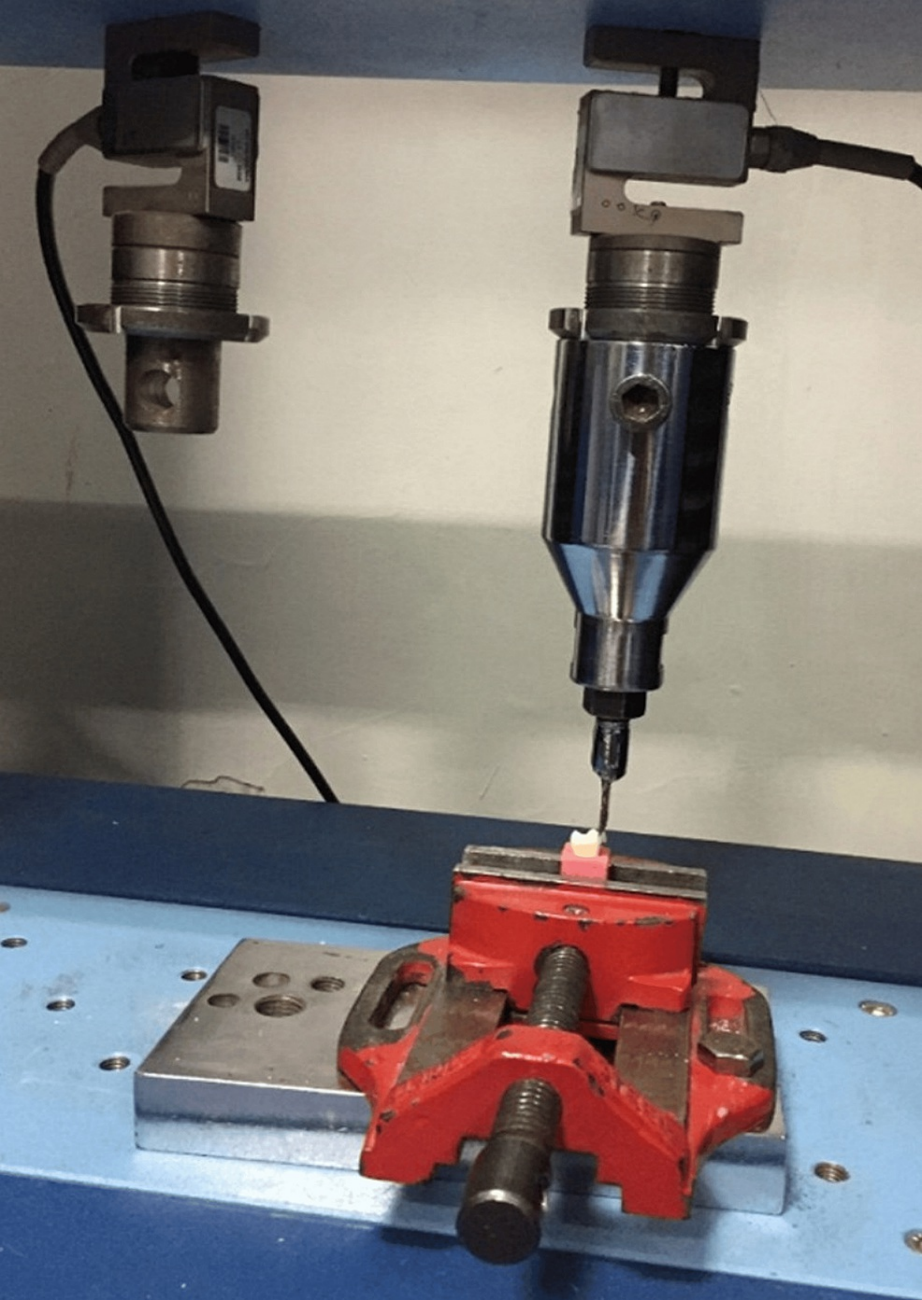
Premolar mounted to test shear bond strength

The bracket was wrapped around a wire that had been fastened to the testing machine's crosshead. Wire loading the bracket with gingivo-occlusal forces caused shear stress at the bracket-tooth contact, which ultimately led to the bracket's debonding. Along with each debonding, a computer linked to the universal testing machine documented the outcome. The necessary force for bracket debonding was detected using an Instron universal testing machine, a computerized universal testing equipment developed by Star Testing Systems, an Indian software business. The machine's crosshead speed was set at 1.0 mm/min, and the findings were recorded in megapascals for each test. The shear bond strength was evaluated using the aforementioned steps (Figure 4).

Adhesive remnant index

**Figure 5:**
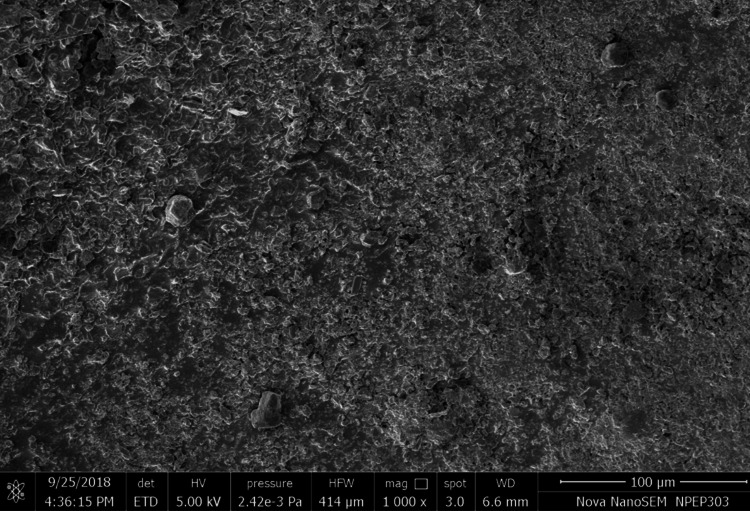
SEM Image for experimental group (APC flash-free brackets) for enamel damage index SEM: Scanning electron microscopy; APC: Adhesive pre-coated

Following the removal of the brackets, the dentist would use the ARI to visually evaluate the surface of each tooth's enamel. The format of the grading procedure was: 1) Glue remained in place even after the bracket's base made imprints on the tooth. There was still adhesive on the tooth in one of five states; 2) More than 90%; 3) Between 10% and 90%; 4) Less than 10%; and 5) No adhesive at all [10]. After enamel microfracture, the teeth were examined visually under a dental operating light to ensure that all of the composite resin had been removed. The teeth were thereafter sprayed with water to clean them. An FEI Nova NanoSEM 450 scanning electron microscope was used to examine the tooth surface sections. Using the enamel damage index, the acquired scanning electron microscopy (SEM) images were analyzed for microfracture in the enamel (Figure 5).

## Results

This study investigated the shear bond strength of two types of brackets-APC flash-free adhesive brackets made by 3M and MBT metal brackets made by AO-in a comparative fashion. A total of 60 samples were taken. Two groups (experimental and control) were made, and 30 samples were distributed in each group. All specimens were subjected to experimentation on a universal testing machine. The readings were recorded and statistically analyzed (Table 1).

**Table 1:** Comparison of shear bond strength among two groups Independent t test; * indicates significant at p≤0.05 APC: Adhesive pre-coated; df: Degrees of freedom

Groups	N	Mean±Std Deviation	Mean Difference	df	T-Value	p-Value	95% CI	
							Minimum	Maximum
Control	30	5.70±2.14	-5.26	58	-4.359	0.001*	-7.71	-2.81
APC	30	10.96±6.26						

An independent t-test comparing the two groups showed a statistically significant difference (P=0.001). Therefore, there was a statistically significant difference in the mean shear bond strengths of the two groups. With a standard deviation of 2.14, the control group demonstrated a mean shear bond strength of 5.70 MPa. With a standard deviation of 6.26, the experimental group had an average shear bond strength of 10.96 MPa.

A visual analog scale was used to assess the enamel surface of each tooth after the brackets were debonded on the universal testing equipment. Based on the ARI, the score was determined (Table 2).

**Table 2:** Comparison of ARI among two groups Independent t-test; * indicates significant at p≤0.05 ARI: Adhesive remnant index; APC: Adhesive pre-coated; df: Degrees of freedom

Groups	N	Mean±Std Deviation	Mean Difference	df	T-Value	p-Value	95% CI	
							Minimum	Minimum
APC	30	2.4±1.07	-0.57	58	-1.995	0.050*	-1.135	-0.002
Control	30	2.97±1.13						

Comparing the two groups using an independent t-test showed that there were statistically significant changes in the mean ARI (P=0.05). Sticker remnant index scores for the control group averaged 2.97 and varied by 1.13 standard deviations. Among the test subjects, the average ARI score was 2.4, with a standard deviation of 1.07.

After debonding the brackets, the enamel microfracture was checked using SEM images. The surface of each tooth was examined using a visual analogue scale, and a score was given according to the enamel damage index (EDI) (Table 3).

**Table 3:** Comparison of EDI among two groups Independent t test; * indicates significant at p≤0.05 EDI: Enamel damage index; APC: Adhesive pre-coated; df: Degrees of freedom

Groups	N	Mean±Std Deviation	Mean Difference	df	T-Value	p-Value	95% CI	
							Minimum	Maximum
Control	30	1.53±0.97	-0.30	58	-1.282	0.205	-0.768	0.169
APC	30	1.83±0.83						

The two groups were compared using independent t-test, showing no statistically significant change with respect to mean ARI (P=0.205). Mean EDI score of the control group was 1.53, with standard deviation of 0.97. Mean EDI score of the experimental group was 1.83, with standard deviation of 0.83.

## Discussion

The evolution from banding to bonding in orthodontic treatment has highlighted the importance of bonding procedures and bonding materials. Regarding bonding materials, shear bond strength plays a prime role in orthodontics. This study found a marked difference between two different bonding systems in shear bond strength, ARI, and enamel microfracture. A wire was looped around the crosshead testing machine’s bracket. A gingival-occlusal tension was applied to each of the 60 brackets using this wire.

Although both types of orthodontic brackets had a high adhesive residual index, Guzman et al. observed that pre-coated orthodontic brackets maintained a higher shear bond strength after debonding than traditionally bonded orthodontic brackets [9]. The bond strength can be maintained at the same level as with a pre-coated device provided the latter is used and the patient is advised to maintain a soft diet. After debonding, the binding strength of the pre-coated object increased quickly [9].

In an in vitro trial, researchers randomly allocated 36 removed human maxillary premolars to one of three groups: APC clarity advance ceramic bracket, APC plus bracket, and a control group with APC plus bracket. Once the pre-coated brackets were glued to the teeth, the occlusal-gingival and mesiodistal positions of all of the teeth were precisely determined. “Transbond XT light cure adhesive paste was used to secure the brackets that were manually inserted. Although all three bracket systems achieved sufficient bond strengths, the APC flash-free adhesive-coated brackets achieved the highest levels of shear bond strength in the shortest amount of time” [10].

Sfondrini et al. looked at gum-changed glass-ionomer concrete in addition to composite tar [11]. Based on his investigation, the designer concluded that the mean shear bond characteristics provided by composite sap and tar-changed glass ionomer concrete are comparable. However, while drawing is performed with 37% phosphoric destructive before holding, the bond characteristics significantly outperform those of tar-modified glass ionomer concrete [11].

Armstrong et al. investigated the presence of white spot lesions surrounding orthodontic brackets, which are often caused by excess adhesive flash during bracket implantation [12]. White spot lesions are most commonly seen around upper lateral incisors, upper canines, and lower premolars. They concluded that clinicians need to be more vigilant. A significant amount of adhesive flash removal will reduce white spot lesions, gingival irritation, and plaque-retentive areas [12].

An SEM analysis of brackets confirmed the presence of mature plaque and extra composite and buccal enamel. According to the findings of this research, plaque builds up rapidly in areas where there is an abundance of composite material, such as around the bracket and bracket base [13]. Using APC streak-free glue pre-coated parts led to bond disappointment at the finish cement interface, whereas using AO metal sections led to disappointment more often at the section glue contact, as the current study showed. There will be far-reaching repercussions of this research. If one part does not work, any of the two joints might cause a whole new set of complications. The veneer surface goes from being low-energy hydrophobic to high-energy hydrophilic during corrosive drawing, which causes a rise in surface strain and wettability. When a bracket breaks at the adhesive contact, there is little enamel microfracturing and strong adhesion. There is a good chance that the enamel surface will be damaged during the cleaning process, but the patient will be stuck in the chair for quite a while if any adhesive remains [14].

Primer was utilized, and the bond strength was satisfactory in the AO brackets (transbond XT). The enamel’s surface was acid etched, resulting in microscopic imperfections that the primer was able to fill. With this method, tiny resin “tags” may be included in the enamel, leading to a micro mechanical interlock between the enamel and resin that, in time, creates sufficient shear bond strength [15]. Ozer et al. discovered that although adhesive failures reduced the amount of adhesive left on the enamel and the amount of time needed to clean the enamel, they also increased the chances of enamel loss [16]. Therefore, cohesive failures are preferred to adhesive failures. Dentists should use metal brackets if possible because ceramic brackets are more likely to crack enamel [16]. Even with a composite resin bonding agent, acid-etched enamel may fracture owing to the micromechanical link between the two [17]. Thus, it is safe to state that each time the link between the adhesive and the enamel fails, some enamel will be lost.

Bond failure in this investigation was shown to occur at the adhesive-enamel contact, suggesting that the APC flash-free bracket system is superior. There can be a slight amount of loss due to micromechanical bonding of the brackets. These results were obtained after analyzing the SEM images of both the control and experimental groups. Both groups showed no significant statistical difference in enamel microfracture, but individually, the enamel microfracture of the APC flash-free brackets had greater microfracture.

The APC flash-free brackets used in this research had strong shear bond strength, which helped prevent debonding and contributed to better treatment outcomes by cutting down on the amount of time spent fixing breaks [18]. As the flash is reduced in this particular appliance system, decreased plaque accumulation and reduction of the amount of white spot lesions are achieved [19]. However, this is an in vitro study for testing shear bond strength. A further simulation can be done by keeping the samples in natural saliva instead of artificial saliva, which will simulate the exact oral environment. Further studies can be done to reduce the enamel microfracture in the experimental group.

This study has certain caveats, the most notable of which being the limited sample size (60 recently removed human premolars). A larger sample size would enhance the generalizability of the findings. Furthermore, the study design focused primarily on laboratory conditions, which may not fully replicate the dynamic oral environment encountered in clinical practice. Additionally, the short-term nature of the study did not assess the long-term durability and performance of the bonded brackets, which is crucial for evaluating the clinical success of orthodontic treatments. Future studies should consider longitudinal follow-ups to assess the stability and longevity of the bond over time. To further understand how the bonding process affects shear bond strength and other important outcomes, future studies should try to account for these confounding factors.

## Conclusions

There was significantly greater shear bond strength obtained by APC flash-free brackets after etching with normal 37% phosphoric acid compared to the control group, which was bonded using a conventional procedure. These results will allow orthodontists to use APC brackets without excessive flash, thus reducing the time for clean-up and enamel demineralization. The APC flash-free brackets debond more frequently at the adhesive-element interface than the conventional bonding system does at the adhesive-bracket interface, as evidenced by a lower ARI compared to the control group. The APC flash-free bracket system had a higher rate of enamel microfracture as compared to the control group. Debonding at the adhesive enamel contact, caused by higher shear bond strength and lower adhesive residual index, might be the reason for this.

## References

[1] Reynolds IR (1975). A review of direct orthodontic bonding. Br J Orthod.

[2] Boersma JG, van der Veen MH, Lagerweij MD, Bokhout B, Prahl-Andersen B (2005). Caries prevalence measured with QLF after treatment with fixed orthodontic appliances: influencing factors. Caries Res.

[3] Sharma P, Valiathan A, Arora A, Agarwal S (2013). A comparative evaluation of the retention of metallic brackets bonded with resin-modified glass ionomer cement under different enamel preparations: a pilot study. Contemp Clin Dent.

[4] Foersch M, Schuster C, Rahimi RK, Wehrbein H, Jacobs C (2016). A new flash-free orthodontic adhesive system: a first clinical and stereomicroscopic study. Angle Orthod.

[5] Grünheid T, Sudit GN, Larson BE (2015). Debonding and adhesive remnant cleanup: an in vitro comparison of bond quality, adhesive remnant cleanup, and orthodontic acceptance of a flash-free product. Eur J Orthod.

[6] Bishara SE, Soliman MM, Oonsombat C, Laffoon JF, Ajlouni R (2004). The effect of variation in mesh-base design on the shear bond strength of orthodontic brackets. Angle Orthod.

[7] Bishara SE, Trulove TS (1990). Comparisons of different debonding techniques for ceramic brackets: an in vitro study: part II. findings and clinical implications. Am J Orthod Dentofacial Orthop.

[8] Alessandri Bonetti G, Zanarini M, Incerti Parenti S, Lattuca M, Marchionni S, Gatto MR (2011). Evaluation of enamel surfaces after bracket debonding: an in-vivo study with scanning electron microscopy. Am J Orthod Dentofacial Orthop.

[9] Guzman UA, Jerrold L, Vig PS, Abdelkarim A (2013). Comparison of shear bond strength and adhesive remnant index between precoated and conventionally bonded orthodontic brackets. Prog Orthod.

[10] Lee M, Kanavakis G (2016). Comparison of shear bond strength and bonding time of a novel flash-free bonding system. Angle Orthod.

[11] Sfondrini MF, Cacciafesta V, Pistorio A, Sfondrini G (2001). Effects of conventional and high-intensity light-curing on enamel shear bond strength of composite resin and resin-modified glass-ionomer. Am J Orthod Dentofacial Orthop.

[12] Armstrong D, Shen G, Petocz P, Darendeliler MA (2007). Excess adhesive flash upon bracket placement. A typodont study comparing APC PLUS and Transbond XT. Angle Orthod.

[13] Sukontapatipark W, el-Agroudi MA, Selliseth NJ, Thunold K, Selvig KA (2001). Bacterial colonization associated with fixed orthodontic appliances. A scanning electron microscopy study. Eur J Orthod.

[14] Newman GV (1965). Epoxy adhesives for orthodontic attachments: progress report. Am J Orthod Dentofacial Orthop.

[15] Britton JC, Mcinnes P, Weinberg R, Ledoux WR, Retief DH (1990). Shear bond strength of ceramic orthodontic brackets to enamel. Am J Orthod Dentofacial Orthop.

[16] Ozer T, Başaran G, Kama JD (2010). Surface roughness of the restored enamel after orthodontic treatment. Am J Orthod Dentofacial Orthop.

[17] Surmont P, Dermaut L, Martens L, Moors M (1992). Comparison in shear bond strength of orthodontic brackets between five bonding systems related to different etching times: an in vitro study. Am J Orthod Dentofacial Orthop.

[18] Bradburn G, Pender N (1992). An in vitro study of the bond strength of two light-cured composites used in the direct bonding of orthodontic brackets to molars. Am J Orthod Dentofacial Orthop.

[19] Damon PL, Bishara SE, Olsen ME, Jakobsen JR (1996). Effects of fluoride application on shear bond strength of orthodontic brackets. Angle Orthod.

